# Candidate Gene Association Analysis of Neuroblastoma in Chinese Children Strengthens the Role of *LMO1*

**DOI:** 10.1371/journal.pone.0127856

**Published:** 2015-06-01

**Authors:** Jie Lu, Ping Chu, Huanmin Wang, Yaqiong Jin, Shujing Han, Wei Han, Jun Tai, Yongli Guo, Xin Ni

**Affiliations:** 1 Beijing Key Laboratory for Pediatric Diseases of Otolaryngology, Head and Neck Surgery, Beijing Pediatric Research Institute, Beijing Children’s Hospital, Capital Medical University, Beijing, China; 2 Department of Surgical Oncology, Beijing Children’s Hospital, Capital Medical University, Beijing, China; 3 Head and Neck Surgery, Beijing Children’s Hospital, Capital Medical University, Beijing, China; Medical College of Soochow University, CHINA

## Abstract

Neuroblastoma (NB) is the most common extra-cranial solid tumor in children and the most frequently diagnosed cancer in the first year of life. Previous genome-wide association studies (GWAS) of Caucasian and African populations have shown that common single nucleotide polymorphisms (SNPs) in several genes are associated with the risk of developing NB, while few studies have been performed on Chinese children. Herein, we examined the association between the genetic polymorphisms in candidate genes and the risk of NB in Chinese children. In total, 127 SNPs in nine target genes, revealed by GWAS studies of other ethnic groups and four related lincRNAs, were genotyped in 549 samples (244 NB patients and 305 healthy controls). After adjustment for gender and age, there were 21 SNPs associated with NB risk at the two-sided *P* < 0.05 level, 11 of which were located in *LMO1*. After correction for multiple comparisons, only rs204926 in *LMO1* remained significantly different between cases and controls (OR = 0.45, 95% CI: 0.31–0.65, adjusted *P *= 0.003). In addition, 16 haplotypes in four separate genes were significantly different between case and control groups at an unadjusted *P* value < 0.05, 11 of which were located in *LMO1*. A major haplotype, ATC, containing rs204926, rs110420, and rs110419, conferred a significant increase in risk for NB (OR = 1.82, 95% CI: 1.41–2.36, adjusted *P* < 0.001). The major finding of our study was obtained for risk alleles within the *LMO1* gene. Our data suggest that genetic variants in *LMO1* are associated with increased NB risk in Chinese children.

## Introduction

Neuroblastoma (NB) is an embryonal tumor derived from neural crest tissues, which can develop anywhere along the sympathetic nervous system. At least half of NB tumors originate in the adrenal medulla, and others commonly occur in paraspinal sympathetic ganglia of the neck, chest, abdomen, or pelvis [1, 2]. NB is a childhood cancer with an incidence of 10.2 cases per million children younger than 15 years in the United States. It is the most frequently diagnosed cancer in the first year of life and the most common extra-cranial solid tumor in children [1, 2]. NB accounts for more than 7% of all cancers in children under 15 years of age and around 15% of all childhood oncology deaths [2]. The presentation of NB has highly variable clinical behaviors, including heterogeneous clinical outcomes and diverse clinical symptoms, ranging from a lack of symptoms to dramatic symptoms such as Horner’s syndrome and cord compression [1, 2]. Infant patients are more likely to have only localized tumors and to be cured without cytotoxic therapy, while older patients often have metastatic disease at the time of diagnosis and are at high risk for death from refractory disease [1]. The clinical heterogeneity indicates the complicated genomic abnormalities in the tumor cells, and thus has led to studies on the genetics and genomics of NB [3].

Based on previous genetic studies, NB cases can be divided into two subgroups, namely, familial NB (< 2% of all NBs) and sporadic NB [4]. The activating mutations in the *ALK* gene (encoding anaplastic lymphoma kinase) and loss-of-function mutations in the *PHOX2B* gene (encoding paired-like homeobox 2B) account for most cases of familial NB [1]. In sporadic NB patients, malignant tumor development may be due to the combined interaction of common individual DNA variants with modest effects on susceptibility [1]. Genome-wide association studies (GWAS) of NB have shown that common single nucleotide polymorphisms (SNPs) in several genes are associated with the risk of developing NB [3]. These genes include *LINC00340* (long intergenic non-protein coding RNA 340) at chromosome 6p22 [5], *BARD1* (*BRCA1*-associated RING domain-1) at chromosome 2q35 [6–9], *LMO1* (LIM domain only 1) at chromosome 11p15 [10], *HACE1* (HECT domain- and ankyrin repeat-containing E3 ubiquitin protein ligase 1) and *LIN28B* (lin-28 homolog B) at 6q16 [7], *DUSP12* (dual-specificity phosphatase 12) at 1q23.3, *DDX4* (DEAD box polypeptide 4 isoform) and *IL31RA* (interleukin-31 receptor A precursor) at 5q11.2, and *HSD17B12* (hydroxysteroid-17-beta dehydrogenase 12) at 11p11.2 [11].

However, GWAS of NB has thus far been limited to Caucasian and African ethnic groups, and little is known about the association between the above-mentioned candidate genes and NB in the Chinese population. In addition, long intergenic non-coding RNA (lincRNA) can regulate gene expression, and recent studies have shown that SNPs in lincRNA are associated with the risk of various cancers [12–14]. Thus, investigation of the association between SNPs from the candidate genes and related lincRNAs in Chinese children would provide important insight into the etiology of NB. Here, we genotyped 127 SNPs in nine candidate genes and their lincRNAs in a sample of 549 Chinese children. The aim of our study was to reveal the candidate genes identified by GWAS studies of other ethnic groups and determine their contribution to the susceptibility of NB in Chinese children.

## Materials and Methods

### Ethics statement

Our research has been conducted according to the principles expressed in the Declaration of Helsinki and approved by the Ethics Committee of the Beijing Children’s Hospital, Capital Medical University (Beijing, China). For children participants aged 8 and above, written informed consents have been obtained from both the children and the guardians. For children participants younger than 8, written informed consents have been obtained from the guardians.

### Study subjects

To investigate the association between candidate genes and NB, 244 patients and 305 control subjects were enrolled in this case-control study. All patients were recruited from inpatients diagnosed with NB at Beijing Children’s Hospital. All controls were children admitted at the hospital for physical examination, with inclusion criteria of no history of cancer and matched to cases by gender.

### Candidate SNP selection

Due to the high cost and large sample requirement of GWAS, we have used computer-facilitated methods to select candidate SNPs, together with targeted SNP genotyping, to investigate the association between candidate genes and risk of NB in Chinese children. In order to select genes, we performed a literature search of GWAS studies. Then we searched for candidate SNPs within each gene using computer-facilitated selection methods with four steps. First, tagging SNPs within each candidate gene were identified from the HapMap database (http://hapmap.ncbi.nlm.nih.gov/) by using Haploview (http://www.broad.mit.edu/mpg/haploview/) with the selected criteria of minor allele frequency > 0.05 in the Chinese population and *r* ^2^ values > 0.8. For each gene, the search for tagging SNPs extended to a 10 kb region (with 5 kb upstream and 5 kb downstream) surrounding the gene. Secondly, we searched for SNPs located within functional regions in the HapMap database and 1000 Genomes database (http://www.1000genomes.org/), using the following selection criteria: (i) SNPs with a minor allele frequency > 0.05 in the Chinese population and (ii) SNPs located in exons, 5′ UTR, promoter, and 3′ UTR of the candidate genes. In the third step, we scanned the possible regulatory regions of candidate genes (with 5000 kb upstream and 5000 kb downstream) to find related lincRNA that might regulate the expression of candidate genes. Finally, target SNPs derived from the GWAS literature were chosen.

### Sample preparation and genotyping

Blood samples from patients and controls were collected and stored at -80°C. Genomic DNA was extracted from 300 μL peripheral blood samples using the QIAamp DNA Blood Midi Kit (Qiagen, Hilden, Germany) and stored at -20°C until use. Genotyping was performed using the MassArray platform, according to the manufacturer’s protocol (Sequenom, San Diego, CA, USA) [15].

### Statistical analysis

Differences in gender between cases and controls were examined by a two-sided *χ* ^*2*^ test using SPSS 19.0 (IBM Corporation, Armonk, New York, USA). Any SNP with a call rate < 90%, MAF < 0.01, or Hardy-Weinberg equilibrium *P* < 0.001 was excluded from the association analysis. The association analysis was performed using PLINK 1.07 [16]. *χ* ^*2*^ tests were used to evaluate differences in the distributions of allele frequencies between cases and controls. The associations between the polymorphisms and risk of NB were estimated by odds ratios (ORs) and their 95% confidence intervals (95% CIs), calculated by using unconditional logistic regression analysis. Bonferroni adjustment was performed to correct for multiple comparisons in the single SNP analysis. Haploview 4.2 [17] was used in linkage disequilibrium (LD) measurement and haplotype analysis. The content of haplotype analysis included estimating of haplotypes, plotting of haplotype block structure and testing of haplotype associations. All statistical tests were two-sided.

## Results

### Baseline clinical characteristics

A total of 244 NB patients and 305 controls were enrolled in this study. The baseline clinical characteristics of the participants are summarized in Table 1. Staging of disease and diagnosis followed INSS criteria [18]. The distribution of sex between cases and controls was not significantly different, as examined by a two-sided *χ* ^*2*^ test. Among the patients, 163 (66.8%) were between 12 to 60 months of age, 34 (13.9%) were less than 12 months and 46 patients (18.9%) were older than 60 months. With regard to clinical stages, 31 patients were in stage I (12.7%), 37 patients were in stage II (15.2%), 66 patients were in stage III (27.0%), 86 patients were in stage IV (35.2%) and 15 patients were in stage 4s (6.2%). Most of the tumors were derived from the abdominal region, including 111 (45.5%) in the adrenal gland, 102 (41.8%) in the retroperitoneal region, 12 (4.9%) in the pelvic cavity, and two (0.8%) in the sacrococcygeal region.

**Table 1:** Distribution of select characteristics among patients and controls.

Variable	Patients	N (%)	Controls	N (%)	*P* value^a^
Gender					0.099
Male	142	58.2%	156	51.1%	
Female	102	41.8%	149	48.9%	
Age					<0.001
<12 months	34	13.9%	1	0.3%	
12–60 months	163	66.8%	33	10.8%	
>60 months	46	18.9%	269	88.2%	
Unknown	1	0.4%	2	0.7%	
Clinical Stage					
I	31	12.7%			
II	37	15.2%			
III	66	27.0%			
IV	86	35.2%			
4s	15	6.1%			
Unknown	9	3.7%			
Site of origin					
Neck	6	2.5%			
Abdomen					
Adrenal gland	111	45.5%			
Retroperitoneal region	102	41.8%			
Pelvic cavity	12	4.9%			
Sacrococcygeal region	2	0.8%			
Unknown	11	4.5%			

^a^Two-sided *χ* ^*2*^ test

### Genes and alleles

After searching the literatures, we selected nine genes (*LINC00340*, *BARD1*, *LMO1*, *HACE1*, *LIN28B*, *DUSP12*, *DDX4*, *IL31RA*, and *HSD17B12*) for analysis. Based on bioinformatics analysis, four lincRNAs (*LINC00577*, *RP11-613D13*.*8*, *RP11-155L15*.*1*, and *RP11-524C21*.*2*) related to these nine genes were also selected, and 127 SNPs on the above genes and lincRNAs were genotyped (Table 2, S1 Table). Among these SNPs, four failed to amplify in 90% of the samples, two were not polymorphic in our sample, and three SNPs were not in Hardy-Weinberg equilibrium in the controls (*P* < 0.001). A total of 118 SNPs were included in the association analysis. Of these, 23 SNPs in four genes showed case-control differences of allelic frequencies at an unadjusted *P* value < 0.05. However, only three SNPs (rs204926, rs110420, and rs110419) showed significant differences between the case and control groups after Bonferroni adjustment (S2 Table). The T allele of rs204926 (OR = 0.38, adjusted *P* = 2.5 × 10^–8^), the C allele of rs110420 (OR = 0.38, adjusted *P* = 4.9 × 10^–4^), and the G allele of rs110419 (OR = 0.38, adjusted *P* = 7.3 × 10^–4^) are all non-risk alleles.

**Table 2:** List of genes, physical location, and number of candidate SNPs.

Gene/LincRNA	Chromosome	Location	Number of candidate SNPs
*BARD1*	2	215301519–215382673	23
*DDX4*	5	55070534–55148362	5
*DUSP12*	1	159986204–159993576	7
*HACE1*	6	104728093–104859919	10
*HSD17B12*	11	43658718–43834745	11
*IL31RA*	5	55183090–55254434	8
*LIN28B*	6	5001–131285	9
*LINC00340*	6	21666444–22194400	3
*LINC00577*	6	105384169–105388402	2
*LMO1*	11	8202432–8246758	26
*RP11-155L15*.*1*	5	55609732–55617224	15
*RP11-524C21*.*2*	6	22221010–22222624	3
*RP11-613D13*.*8*	11	43965337–43968756	5
		Total	127

### Single SNP analysis

**Fig 1:**
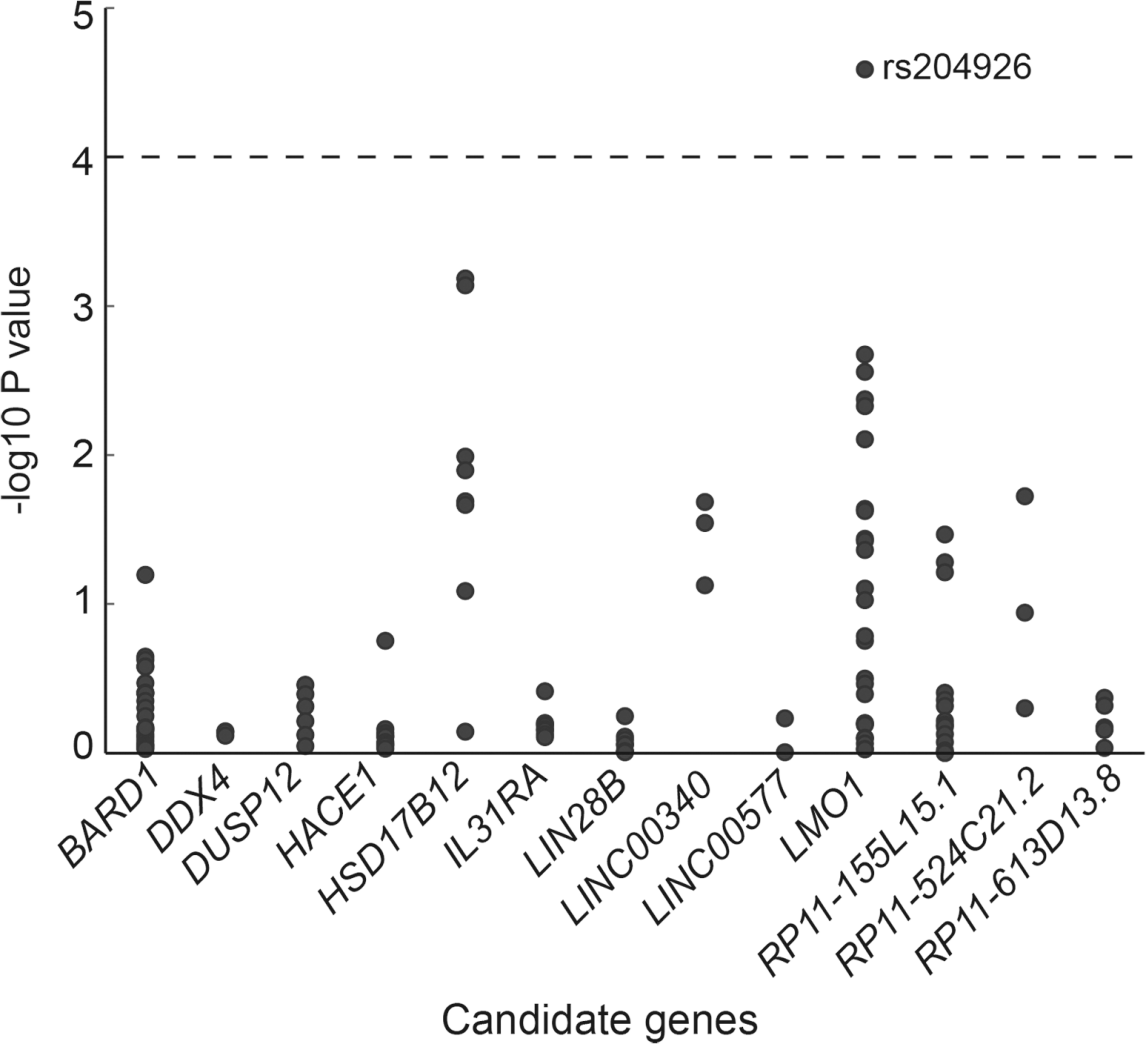
Association results from 127 SNPs using the additive model. For each SNP in the genes shown on the x-axis, the-log10 *P* (unadjusted *P* value) from the logistic regression analysis is indicated on the y-axis. The dashed horizontal line represents the significance threshold (*P* < 0.0001).

To estimate the association between the genotype and risk of NB, we performed logistic regression analysis for all SNPs in this report. Since the underlying genetic model for NB is unknown, the additive model was used for the analysis. Without any adjustment, 22 SNPs in *LMO1* (14), *HSD17B12* (3), *BARD1* (3), and *LINC00340* (2) showed significant case-control differences (S3 Table). After adjustment for gender and age, 21 SNPs in five genes were associated with NB risk (*P* < 0.05, two-sided; Fig 1, Table 3). Out of these 21 SNPs, 11 were located in *LMO1*, and six were found in *HSD17B12*. After Bonferroni adjustment, the only statistically significant case-control difference observed was for rs204926 in *LMO1* (*P* = 0.003). The OR of the T allele of rs204926 for NB was 0.45 (95% CI: 0.31–0.65).

**Table 3:** Risk alleles associated with neuroblastoma in Chinese children revealed by logistic regression analysis with adjustment for gender and age.

SNP	Gene	Chromosome position	A1/A2^a^	OR (95%CI)	*P*	Adjusted *P*
**rs204926**	***LMO1***	8255106	**T/C**	**0.45 (0.31–0.65)**	**<0.0001**	**0.003** ^**b**^
rs10838184	*HSD17B12*	43869860	C/G	0.41 (0.24–0.68)	0.001	0.078
rs11037575	*HSD17B12*	43728330	T/C	0.50 (0.33–0.75)	0.001	0.086
rs2290451	*LMO1*	8248440	C/G	1.91 (1.26–2.89)	0.002	0.252
rs110420	*LMO1*	8253049	C/T	0.61 (0.44–0.84)	0.003	0.328
rs110419	*LMO1*	8252853	G/A	0.62 (0.45–0.86)	0.004	0.502
rs11041816	*LMO1*	8243798	G/A	0.48 (0.29–0.80)	0.005	0.557
rs204938	*LMO1*	8278197	G/A	1.67 (1.14–2.43)	0.008	0.932
rs11606658	*HSD17B12*	43795420	T/C	0.61 (0.42–0.89)	0.010	1.000
rs1061810	*HSD17B12*	43877934	A/C	0.60 (0.40–0.90)	0.013	1.000
rs1980433	*RP11-524C21*.*2*	22114113	G/A	1.48 (1.07–2.04)	0.019	1.000
rs7115970	*HSD17B12*	43769295	C/T	0.62 (0.41–0.93)	0.020	1.000
rs6939340	*LINC00340*	22140004	A/G	0.67 (0.48–0.94)	0.021	1.000
rs11555762	*HSD17B12*	43876698	T/C	0.61 (0.40–0.93)	0.022	1.000
rs4237769	*LMO1*	8275127	A/G	0.69 (0.51–0.95)	0.023	1.000
rs3794012	*LMO1*	8270244	G/A	0.70 (0.51–0.95)	0.024	1.000
rs9295536	*LINC00340*	22131929	C/A	0.67 (0.46–0.96)	0.028	1.000
rs152315	*RP11-155L15*.*1*	55581424	C/T	0.49 (0.26–0.95)	0.034	1.000
rs379951	*LMO1*	8272605	A/G	1.62 (1.03–2.54)	0.036	1.000
rs417210	*LMO1*	8269405	G/T	1.40 (1.02–1.92)	0.038	1.000
rs484161	*LMO1*	8264525	T/C	1.46 (1.01–2.11)	0.043	1.000

^a^ Minor allele/major allele

^b^ Significant results after multiple correction are in bold

### Haplotype analysis

To determine whether joint effects of SNPs had significant risk associations in our samples, we next measured the pair-wise linkage disequilibrium between the SNPs. The SNPs in our study were located on five chromosomes, namely Chromosomes 1, 2, 5, 6 and 11, and we identified several haplotypes using Haploview 4.2. Analyses using a two-sided *χ* ^*2*^ test indicated that the frequencies of 16 haplotypes in four genes showed significant differences between the case and control groups (unadjusted *P* <0.05, S4 Table). After Bonferroni adjustment, only two haplotypes were observed to be significantly different between cases and controls: GCGCT (rs2290451, rs4758053, rs110419, rs110420, and rs204926) and ATGGG (rs4758051, rs7109806, rs11041815, rs12576570, and rs10840002), both in *LMO1*. The GCGCT haplotype contained the statistically significant risk SNP rs204926 (S1 Fig).

In order to reveal whether the joint effects existed in the block containing rs204926, we further analyzed the linkage disequilibrium and haplotype for SNPs only in that block. As shown in S1 Fig, perfect LD (D′ = 1) was observed between several pairs of SNPs, but the *r* ^*2*^ is low (< 0.4). High LDs were found in rs204926, rs110420, and rs110419 (D′ > 0.97, *r* ^*2*^ > 0.59). A major haplotype ATC (66.0%) of the combination of rs204926, rs110420, and rs110419 showed a significant increase in risk for NB (OR = 1.82, 95% CI: 1.41–2.36, *P* < 0.001, Table 4), while another haplotype GCT (23.9%) of the combination of the three SNPs showed a significant decrease in risk for NB (OR = 0.38, 95% CI: 0.28–0.51, *P* < 0.001, Table 4).

**Table 4:** Risk haplotypes associated with neuroblastoma in Chinese children.

Haplotype	Frequency	No. in case (%)	No. in control (%)	OR (95% CI)	*P*	Adjusted *P*
ATC	66.0%	358 (73.3%)	367 (60.1%)	1.82 (1.41–2.36)	4.4 × 10^–6^	1.9 × 10^–4^
GCT	23.9%	72 (14.7%)	191 (31.3%)	0.38 (0.28–0.51)	1.7 × 10^–10^	7.3 × 10^–9^
GCC	9.7%	57 (11.7%)	49 (8.1%)	1.51 (1.01–2.26)	0.042	1.000

## Discussion

This study investigated the association between the genetic polymorphisms in candidate genes and the risk of NB in Chinese children. To estimate risk conferred by individual SNPs and/or SNP-SNP interactions, 127 SNPs in nine selected genes revealed by GWAS studies of other ethnic groups and four related lincRNAs were genotyped in 549 samples (244 NB patients and 305 healthy controls). To the best of our knowledge, this was the first study to investigate the association of SNPs in those selected genes and their corresponding lincRNAs with the risk of NB in Han Chinese children. No case of thoracic region tumor was identified in our cohort of Chinese children, which was quite different from the western populations with a large percentage of thoracic neuroblastoma[19]. The possible reasons were ethnic differences between Chinese population and western population as well as only one hospital origin of our cohort.

The major finding of this study was obtained for risk alleles within the *LMO1* gene. The *LMO1* gene encodes an intertwining LIM-only transcriptional regulator and has three paralogues, namely *LMO2*, *LMO3*, and *LMO4* [19]. Previous studies have shown that *LMO1* is involved in the regulatory network for nervous system development [20–22] and the *LMO1* promoter can enhance gene expression in the developing central nervous system [23], while combined null mutation of the *LMO1*/*LMO3* genes causes perinatal lethality in mice [24].

A recent study by Wang et al. has identified *LMO1* as a neuroblastoma oncogene [10]. That study used four cohorts and identified five significant SNPs associated with NB, namely rs4758051 (G allele; OR = 1.30, *P* = 6.94 × 10^–11^), rs10840002 (A allele; OR = 1.24, *P* = 4.03 × 10^–7^), rs110419 (G allele; OR = 0.74, *P* = 1.35 × 10^–13^), rs204938 (C allele; OR = 1.20, *P* = 1.79 × 10^–6^), and rs110420 (C allele; OR = 0.74, *P* = 1.35 × 10^–13^). The above-mentioned SNPs were all genotyped in our study. Among these SNPs, only rs110419 (G allele; OR = 0.62, *P* = 0.004), rs110420 (C allele; OR = 0.61, *P* = 0.003), and rs204938 (C allele; OR = 1.67, *P* = 0.008) were significantly associated with NB risk at the two-sided *P* < 0.05 level after adjustment for gender and age; none remained significant after correction for multiple comparisons. The trends of previous studies and our study are similar, but the exact results vary for several reasons. First, the 244 patients and 305 controls might not represent all of the NB patients and controls in China due to the limited sample size and sample collection location. Second, the genetic characteristics of the Chinese population are different from the European ethnic population, leading to different allele frequencies in these two populations.

The only statistically significant SNP in our study, rs204926, was in high LD (D′ > 0.95, *r* ^*2*^ > 0.60) with rs110420 and rs110419 and a major haplotype, ATC, containing rs204926, rs110420, and rs110419, showed a significant increase in risk for NB (OR = 1.82, adjusted *P* < 0.001).Although the three SNPs are all located in an intronic region, the study by Wang et al. [10] has shown an association between the risk allele A of rs110419 and increased *LMO1* expression and the high expression of *LMO1* promotes cell proliferation. In consideration of the important role of LMO1 in the nervous system development, it is possible that the above risk alleles (A of rs110419, T of rs110420, and C of rs204926) and haplotype (ATC) would lead to increased *LMO1* expression, and thus higher risk of NB in Chinese children.

Of interest is the fact that we observed a trend toward significance for two SNPs in the *HSD17B12* gene, namely rs10838184 and rs11037575 (Fig 1, Table 3). The *HSD17B12* gene encodes human 17 beta-hydroxysteroid dehydrogenase type 12, an enzyme involved in lipid metabolism [25]. Previous studies revealed that *HSD17B12* could be a prognosis marker for several human cancers [26–28]. A recent study by Nguyễn et al. showed that SNPs in *HSD17B12* were associated with low-risk NBs, and rs11037575 was the most significant SNP in the *HSD17B12* gene [11]. The most significant finding in our study was for rs10838184, presenting in moderate LD with rs11037575 (D′ = 1, *r* ^*2*^ = 0.49). Our results indicated that the *HSD17B12* gene might be a risk gene for NB patients in China.

In addition, it is noteworthy that rs6939340 and rs9295536 in *LINC00340*, rs1980433 in *RP11-524C21*.*2*, and rs152315 in *RP11-155L15*.*1* showed significance at the two-sided *P* < 0.05 level after adjustment for gender and age. These results suggested lincRNA might be related to NB risk, which should be confirmed by further study with a larger sample size and possible functional experiments.

Moreover, previous genetic association analysis of clinical NB phenotypes showed that other SNPs at other genes such as rs6435862 and rs7585356 at *BARD1* conferred an increased risk for NB in Caucasian and African patients [7, 8]. In our study, only one SNP in *LMO1* showed a statistically significant association with NB risk. We hypothesize that this lack of association to the other SNPs is due to ethnic differences between the Chinese population and the other populations as well as insufficient statistical power because of the limited sample size. Further studies would be warranted to perform a similar analysis using a larger sample size to increase statistical power.

The average age of healthy children in this study was 6.9 years, much higher than that of patients (mostly younger than 5 years). NB is a malignancy of early childhood in which the average age of morbidity is about 18 months, and most cases are diagnosed before the age of 5 years. Employing older children as healthy controls is more reasonable because there is a lower probability for them to develop the disease, although the mismatch factor might have an influence on the data analysis. If possible, control group of children with matched age and gender should be used in further study.

## Supporting Information

S1 Fig A linkage disequilibrium (LD) plot of *LMO1* constructed in Haploview. (TIF)

S1 Table Summary of candidate SNPs. (DOCX)

S2 Table Significant case-control allele frequency differences examined by two-sided *χ* ^*2*^ test. (DOCX)

S3 Table Significant SNPs associated with neuroblastoma in Chinese children revealed by logistic regression analysis without adjustment of gender and age. (DOCX)

S4 Table Significant case-control haplotype frequency differences examined by two-sided *χ* ^*2*^ test. (DOCX)
