# Supplementary figures and images for: Candidate Gene Association Analysis of Neuroblastoma in Chinese Children Strengthens the Role of LMO1
Source: PLoS One. 2015 Jun 1;10(6):e0127856. doi: 10.1371/journal.pone.0127856 (PMC4452511; doi:10.1371/journal.pone.0127856)

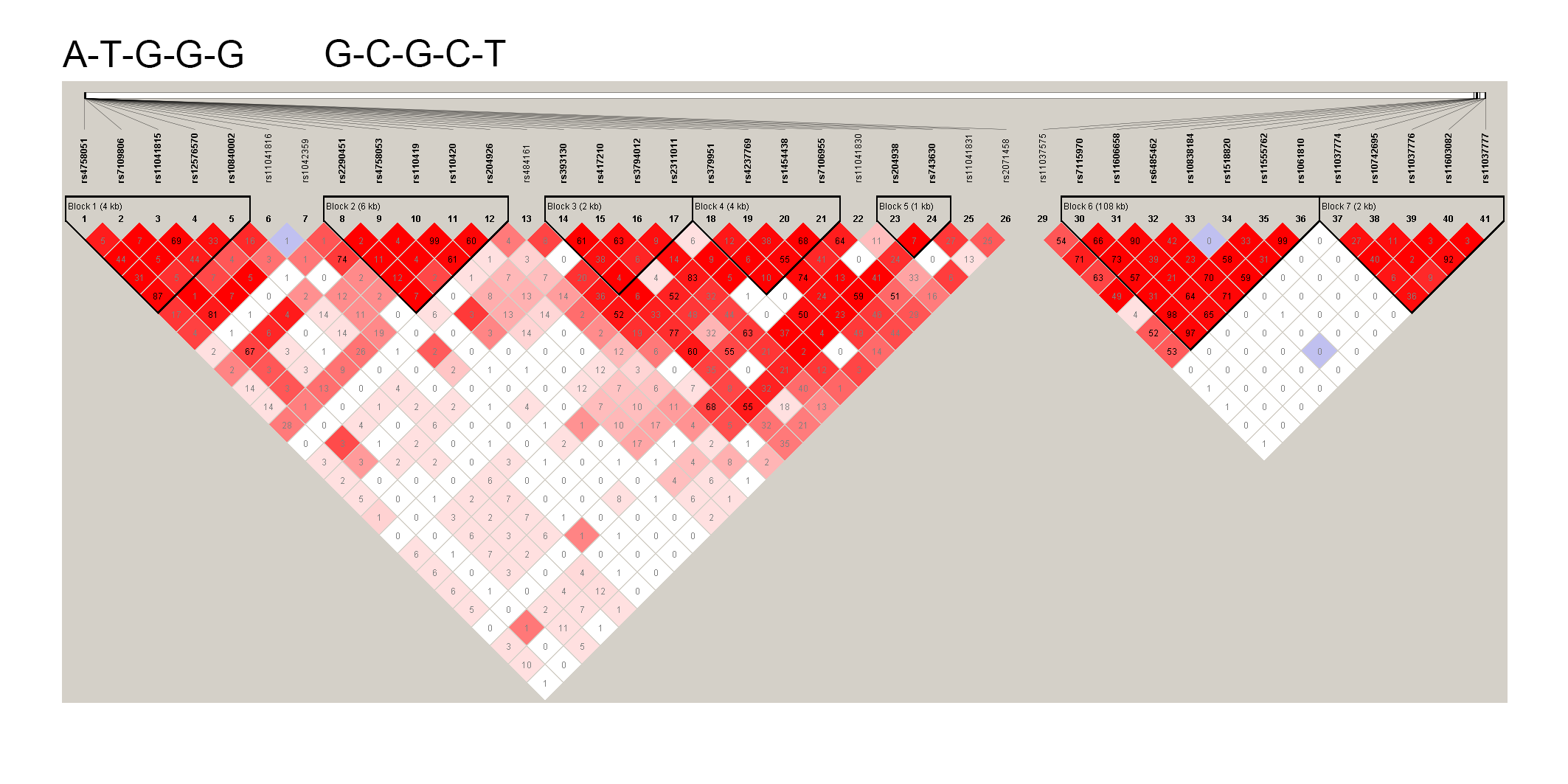

Supplement: S1 Fig — (TIF) [file pone.0127856.s001.tif]
