# Supplementary material for: Candidate Gene Association Analysis of Neuroblastoma in Chinese Children Strengthens the Role of LMO1
Source: PLoS One. 2015 Jun 1;10(6):e0127856. doi: 10.1371/journal.pone.0127856 (PMC4452511; doi:10.1371/journal.pone.0127856)
Supplement: S1 Table — (DOCX) [file pone.0127856.s002.docx]

**S1 Table.** Summary of candidate SNPs.

| SNP | Gene | Chromosome | Position | MAF | A1(Minor allele) | A2 |
| --- | --- | --- | --- | --- | --- | --- |
| rs7585356 | *BARD1* | 2 | 215592306 | 0.32 | A | G |
| rs2075623 | *BARD1* | 2 | 215593851 | 0.42 | C | G |
| rs3738888 | *BARD1* | 2 | 215595164 | 0.04 | A | G |
| rs16852600 | *BARD1* | 2 | 215595645 | 0.32 | T | C |
| rs6759222 | *BARD1* | 2 | 215620757 | 0.09 | A | C |
| rs7599060 | *BARD1* | 2 | 215631302 | 0.34 | A | G |
| rs2070094 | *BARD1* | 2 | 215632255 | 0.25 | T | C |
| rs2070093 | *BARD1* | 2 | 215632256 | 0.12 | A | G |
| rs3768716 | *BARD1* | 2 | 215635794 | 0.15 | G | A |
| rs17487792 | *BARD1* | 2 | 215643500 | 0.13 | T | C |
| rs3768707 | *BARD1* | 2 | 215645135 | 0.19 | A | G |
| rs2229571 | *BARD1* | 2 | 215645464 | 0.34 | G | C |
| rs2070096 | *BARD1* | 2 | 215645545 | 0.13 | C | G |
| rs7566806 | *BARD1* | 2 | 215646728 | 0.47 | G | C |
| rs7587476 | *BARD1* | 2 | 215653887 | 0.19 | T | C |
| rs6712055 | *BARD1* | 2 | 215666904 | 0.42 | T | C |
| rs16852775 | *BARD1* | 2 | 215667360 | 0.07 | C | T |
| rs6435862 | *BARD1* | 2 | 215672546 | 0.12 | G | T |
| rs6715570 | *BARD1* | 2 | 215673440 | 0.18 | T | C |
| rs1048108 | *BARD1* | 2 | 215674224 | 0.37 | A | G |
| rs1129804 | *BARD1* | 2 | 215674323 | 0.18 | C | G |
| rs17489363 | *BARD1* | 2 | 215674341 | 0.18 | A | G |
| rs34732883 | *BARD1* | 2 | 215674371 | 0.18 | C | T |
| rs57335149 | *DDX4* | 5 | 55032014 | 0.32 | T | G |
| rs3789193 | *DDX4* | 5 | 55033768 | 0.32 | G | C |
| rs13181170 | *DDX4* | 5 | 55062764 | 0.44 | C | T |
| rs2305123 | *DDX4* | 5 | 55081694 | 0.12 | G | A |
| rs2619046 | *DDX4* | 5 | 55097534 | 0.46 | A | G |
| rs1503814 | *DUSP12* | 1 | 161709119 | 0.31 | T | C |
| rs1027702 | *DUSP12* | 1 | 161712857 | 0.33 | C | T |
| rs10917835 | *DUSP12* | 1 | 161717293 | 0.34 | A | T |
| rs6427625 | *DUSP12* | 1 | 161718740 | 0.33 | C | G |
| rs1063179 | *DUSP12* | 1 | 161722204 | 0.22 | T | C |
| rs12745240 | *DUSP12* | 1 | 161728001 | 0.44 | A | G |
| rs12121310 | *DUSP12* | 1 | 161719215 | 0.33 | C | A |
| rs9391227 | *HACE1* | 6 | 105158634 | 0.43 | A | T |
| rs45521835 | *HACE1* | 6 | 105176528 | 0.18 | A | G |
| rs4336470 | *HACE1* | 6 | 105180785 | 0.23 | T | C |
| rs6937432 | *HACE1* | 6 | 105191527 | 0.23 | T | C |
| rs9499954 | *HACE1* | 6 | 105205826 | 0.23 | T | C |
| rs9322817 | *HACE1* | 6 | 105232233 | 0.22 | C | A |
| rs12212729 | *HACE1* | 6 | 105281212 | 0.08 | T | C |
| rs6927608 | *HACE1* | 6 | 105288125 | 0.18 | C | A |
| rs2486143 | *HACE1* | 6 | 105308593 | 0.40 | A | G |
| rs6911031 | *HACE1* | 6 | 105311616 | 0.16 | T | C |
| rs6485444 | *HSD17B12* | 11 | 43700194 | 0.13 | C | T |
| rs4755733 | *HSD17B12* | 11 | 43700529 | 0.08 | T | C |
| rs11037575 | *HSD17B12* | 11 | 43728330 | 0.24 | T | C |
| rs7115970 | *HSD17B12* | 11 | 43769295 | 0.21 | C | T |
| rs11606658 | *HSD17B12* | 11 | 43795420 | 0.29 | T | C |
| rs6485462 | *HSD17B12* | 11 | 43816200 | 0.26 | C | T |
| rs10838184 | *HSD17B12* | 11 | 43869860 | 0.11 | C | G |
| rs1518820 | *HSD17B12* | 11 | 43873539 | 0.09 | A | C |
| rs11555762 | *HSD17B12* | 11 | 43876698 | 0.20 | T | C |
| rs1061810 | *HSD17B12* | 11 | 43877934 | 0.20 | A | C |
| rs11037662 | *HSD17B12* | 11 | 43851308 | 0.39 | G | T |
| rs12521422 | *IL31RA* | 5 | 55145019 | 0.12 | G | A |
| rs6872635 | *IL31RA* | 5 | 55147940 | 0.44 | A | T |
| rs9292101 | *IL31RA* | 5 | 55150204 | 0.44 | T | G |
| rs1009639 | *IL31RA* | 5 | 55155402 | 0.44 | T | C |
| rs10055201 | *IL31RA* | 5 | 55161102 | 0.44 | G | A |
| rs161704 | *IL31RA* | 5 | 55206444 | 0.38 | A | G |
| rs11956465 | *IL31RA* | 5 | 55207550 | 0.47 | C | T |
| rs161699 | *IL31RA* | 5 | 55213023 | 0.14 | C | T |
| rs7759938 | *LIN28B* | 6 | 105378954 | 0.27 | C | T |
| rs314263 | *LIN28B* | 6 | 105392745 | 0.27 | C | T |
| rs314280 | *LIN28B* | 6 | 105400837 | 0.27 | A | G |
| rs314276 | *LIN28B* | 6 | 105407999 | 0.27 | A | C |
| rs369065 | *LIN28B* | 6 | 105444058 | 0.36 | C | T |
| rs221634 | *LIN28B* | 6 | 105528088 | 0.48 | A | T |
| rs221635 | *LIN28B* | 6 | 105529674 | 0.20 | C | T |
| rs221636 | *LIN28B* | 6 | 105529964 | 0.20 | T | A |
| rs4145418 | *LIN28B* | 6 | 105529785 | 0.32 | C | A |
| rs4712653 | *LINC00340* | 6 | 22125964 | 0.27 | T | C |
| rs9295536 | *LINC00340* | 6 | 22131929 | 0.28 | C | A |
| rs6939340 | *LINC00340* | 6 | 22140004 | 0.33 | A | G |
| rs6571212 | *linc00577* | 6 | 105277595 | 0.22 | T | A |
| rs1316908 | *linc00577* | 6 | 105278426 | 0.18 | T | C |
| rs4758051 | *LMO1* | 11 | 8238639 | 0.49 | A | G |
| rs7109806 | *LMO1* | 11 | 8242629 | 0.07 | C | T |
| rs11041815 | *LMO1* | 11 | 8242864 | 0.39 | T | G |
| rs12576570 | *LMO1* | 11 | 8243002 | 0.28 | A | G |
| rs10840002 | *LMO1* | 11 | 8243026 | 0.49 | G | A |
| rs11041816 | *LMO1* | 11 | 8243798 | 0.16 | G | A |
| rs1042359 | *LMO1* | 11 | 8246181 | 0.07 | G | A |
| rs2290451 | *LMO1* | 11 | 8248440 | 0.13 | C | G |
| rs4758053 | *LMO1* | 11 | 8249742 | 0.05 | T | C |
| rs110419 | *LMO1* | 11 | 8252853 | 0.50 | G | A |
| rs110420 | *LMO1* | 11 | 8253049 | 0.48 | C | T |
| rs204926 | *LMO1* | 11 | 8255106 | 0.37 | T | C |
| rs484161 | *LMO1* | 11 | 8264525 | 0.20 | T | C |
| rs393130 | *LMO1* | 11 | 8268949 | 0.29 | A | G |
| rs417210 | *LMO1* | 11 | 8269405 | 0.39 | G | T |
| rs3794012 | *LMO1* | 11 | 8270244 | 0.47 | G | A |
| rs2311011 | *LMO1* | 11 | 8271723 | 0.08 | A | G |
| rs379951 | *LMO1* | 11 | 8272605 | 0.13 | A | G |
| rs4237769 | *LMO1* | 11 | 8275127 | 0.50 | A | G |
| rs1454438 | *LMO1* | 11 | 8276249 | 0.29 | T | A |
| rs7106955 | *LMO1* | 11 | 8276698 | 0.36 | G | A |
| rs11041830 | *LMO1* | 11 | 8277807 | 0.42 | A | C |
| rs204938 | *LMO1* | 11 | 8278197 | 0.19 | G | A |
| rs743630 | *LMO1* | 11 | 8280039 | 0.20 | T | C |
| rs11041831 | *LMO1* | 11 | 8282536 | 0.40 | C | G |
| rs2071458 | *LMO1* | 11 | 8285124 | 0.26 | A | C |
| rs2342888 | *RP11-155L15.1* | 5 | 55574191 | 0.48 | G | A |
| rs1862524 | *RP11-155L15.1* | 5 | 55575355 | 0.38 | G | A |
| rs152318 | *RP11-155L15.1* | 5 | 55575558 | 0.45 | A | C |
| rs6450377 | *RP11-155L15.1* | 5 | 55575731 | 0.45 | A | G |
| rs158638 | *RP11-155L15.1* | 5 | 55576016 | 0.40 | A | G |
| rs158487 | *RP11-155L15.1* | 5 | 55576026 | 0.06 | A | G |
| rs158592 | *RP11-155L15.1* | 5 | 55576562 | 0.44 | A | T |
| rs4700208 | *RP11-155L15.1* | 5 | 55578025 | 0.46 | T | C |
| rs149490 | *RP11-155L15.1* | 5 | 55579262 | 0.06 | G | A |
| rs158497 | *RP11-155L15.1* | 5 | 55580137 | 0.41 | G | A |
| rs150364 | *RP11-155L15.1* | 5 | 55580776 | 0.41 | A | G |
| rs158499 | *RP11-155L15.1* | 5 | 55580949 | 0.06 | T | C |
| rs150365 | *RP11-155L15.1* | 5 | 55581028 | 0.06 | A | G |
| rs152315 | *RP11-155L15.1* | 5 | 55581424 | 0.06 | C | T |
| rs150372 | *RP11-155L15.1* | 5 | 55581399 | 0.06 | T | A |
| rs12211235 | *RP11-524C21.2* | 6 | 22113120 | 0.07 | G | A |
| rs9295534 | *RP11-524C21.2* | 6 | 22113390 | 0.27 | T | A |
| rs1980433 | *RP11-524C21.2* | 6 | 22114113 | 0.48 | G | A |
| rs11037774 | *RP11-613D13.8* | 11 | 44009414 | 0.49 | A | G |
| rs10742695 | *RP11-613D13.8* | 11 | 44009451 | 0.16 | G | A |
| rs11037776 | *RP11-613D13.8* | 11 | 44011010 | 0.28 | T | C |
| rs11603082 | *RP11-613D13.8* | 11 | 44011375 | 0.08 | T | A |
| rs11037777 | *RP11-613D13.8* | 11 | 44011969 | 0.28 | A | G |
