# Supplementary material for: Candidate Gene Association Analysis of Neuroblastoma in Chinese Children Strengthens the Role of LMO1
Source: PLoS One. 2015 Jun 1;10(6):e0127856. doi: 10.1371/journal.pone.0127856 (PMC4452511; doi:10.1371/journal.pone.0127856)
Supplement: S2 Table — (DOCX) [file pone.0127856.s003.docx]

**S2 Table.** Significant case-control allele frequency differences examined by two-sided χ^2^ test.

| SNP | Gene | A1/A2 | A1 frequency in case | A1 frequency in control | OR (95% CI) | *P* | Adjusted *P* |
| --- | --- | --- | --- | --- | --- | --- | --- |
| rs204926 | *LMO1* | T/C | 15.0% | 31.6% | 0.38 (0.28-0.52) | 2.1×10^-10^ | 2.5×10^-8^ |
| rs110420 | *LMO1* | C/T | 26.4% | 39.7% | 0.55 (0.42-0.71) | 4.1×10^-6^ | 4.9×10^-4^ |
| rs110419 | *LMO1* | G/A | 26.4% | 39.5% | 0.55 (0.43-0.71) | 6.1×10^-6^ | 7.3×10^-4^ |
| rs4758051 | *LMO1* | A/G | 32.0% | 41.5% | 0.66 (0.52-0.85) | 0.001 | 0.145 |
| rs11041816 | *LMO1* | G/A | 8.2% | 14.2% | 0.54 (0.36-0.80) | 0.002 | 0.238 |
| rs11037575 | *HSD17B12* | T/C | 16.4% | 24.0% | 0.62 (0.46-0.84) | 0.002 | 0.247 |
| rs3794012 | *LMO1* | G/A | 43.7% | 53.0% | 0.69 (0.54-0.87) | 0.002 | 0.256 |
| rs10838184 | *HSD17B12* | C/G | 7.8% | 13.8% | 0.53 (0.35-0.81) | 0.003 | 0.303 |
| rs6939340 | *LINC00340* | A/G | 25.4% | 33.7% | 0.67 (0.52-0.87) | 0.003 | 0.370 |
| rs10840002 | *LMO1* | G/A | 35.1% | 43.9% | 0.69 (0.54-0.88) | 0.003 | 0.384 |
| rs4237769 | *LMO1* | A/G | 42.2% | 50.8% | 0.71 (0.55-0.90) | 0.004 | 0.516 |
| rs417210 | *LMO1* | G/T | 45.5% | 37.5% | 1.39 (1.09-1.78) | 0.007 | 0.884 |
| rs379951 | *LMO1* | A/G | 16.8% | 11.1% | 1.62 (1.13-2.31) | 0.008 | 0.908 |
| rs12576570 | *LMO1* | A/G | 38.9% | 31.4% | 1.40 (1.09-1.79) | 0.009 | 1.000 |
| rs204938 | *LMO1* | G/A | 26.4% | 19.9% | 1.45 (1.09-1.92) | 0.011 | 1.000 |
| rs484161 | *LMO1* | T/C | 25.1% | 18.8% | 1.45 (1.09-1.94) | 0.011 | 1.000 |
| rs2290451 | *LMO1* | C/G | 22.2% | 16.2% | 1.48 (1.08-2.02) | 0.014 | 1.000 |
| rs6435862 | *BARD1* | G/T | 17.7% | 12.5% | 1.51 (1.08-2.10) | 0.016 | 1.000 |
| rs11606658 | *HSD17B12* | T/C | 21.0% | 26.6% | 0.73 (0.55-0.97) | 0.030 | 1.000 |
| rs3768716 | *BARD1* | G/A | 21.6% | 16.5% | 1.40 (1.03-1.90) | 0.031 | 1.000 |
| rs2070096 | *BARD1* | C/G | 22.0% | 17.0% | 1.38 (1.02-1.86) | 0.036 | 1.000 |
| rs9295536 | *LINC00340* | C/A | 20.9% | 26.3% | 0.74 (0.56-0.99) | 0.040 | 1.000 |
| rs6715570 | *BARD1* | T/C | 22.1% | 17.3% | 1.36 (1.01-1.84) | 0.044 | 1.000 |
