# Supplementary material for: Candidate Gene Association Analysis of Neuroblastoma in Chinese Children Strengthens the Role of LMO1
Source: PLoS One. 2015 Jun 1;10(6):e0127856. doi: 10.1371/journal.pone.0127856 (PMC4452511; doi:10.1371/journal.pone.0127856)
Supplement: S3 Table — (DOCX) [file pone.0127856.s004.docx]

**S3 Table.** Significant SNPs associated with neuroblastoma in Chinese children revealed by logistic regression analysis without adjustment of gender and age.

| SNP | Gene | A1/A2^a^ | OR | L95 | U95 | *P* | Adjusted *P* |
| --- | --- | --- | --- | --- | --- | --- | --- |
| **rs204926** | ***LMO1*** | **T/C** | **0.39** | **0.29** | **0.53** | **< 0.001** | **< 0.001^b^** |
| **rs110420** | ***LMO1*** | **C/T** | **0.54** | **0.42** | **0.71** | **< 0.001** | **0.001^b^** |
| **rs110419** | ***LMO1*** | **G/A** | **0.55** | **0.42** | **0.71** | **< 0.001** | **0.001^b^** |
| rs4758051 | *LMO1* | A/G | 0.66 | 0.51 | 0.85 | 0.001 | 0.202 |
| rs3794012 | *LMO1* | G/A | 0.68 | 0.53 | 0.87 | 0.002 | 0.311 |
| rs11037575 | *HSD17B12* | T/C | 0.61 | 0.45 | 0.84 | 0.002 | 0.330 |
| rs11041816 | *LMO1* | G/A | 0.54 | 0.36 | 0.80 | 0.002 | 0.370 |
| rs10838184 | *HSD17B12* | C/G | 0.53 | 0.35 | 0.81 | 0.003 | 0.511 |
| rs6939340 | *LINC00340* | A/G | 0.67 | 0.51 | 0.87 | 0.003 | 0.520 |
| rs4237769 | *LMO1* | A/G | 0.69 | 0.54 | 0.89 | 0.004 | 0.595 |
| rs10840002 | *LMO1* | G/A | 0.70 | 0.55 | 0.89 | 0.004 | 0.670 |
| rs417210 | *LMO1* | G/T | 1.40 | 1.10 | 1.80 | 0.007 | 1.000 |
| rs379951 | *LMO1* | A/G | 1.63 | 1.14 | 2.34 | 0.008 | 1.000 |
| rs204938 | *LMO1* | G/A | 1.48 | 1.10 | 1.99 | 0.009 | 1.000 |
| rs484161 | *LMO1* | T/C | 1.45 | 1.09 | 1.94 | 0.012 | 1.000 |
| rs2290451 | *LMO1* | C/G | 1.51 | 1.09 | 2.08 | 0.012 | 1.000 |
| rs12576570 | *LMO1* | A/G | 1.36 | 1.07 | 1.74 | 0.013 | 1.000 |
| rs6435862 | *BARD1* | G/T | 1.51 | 1.08 | 2.11 | 0.017 | 1.000 |
| rs11606658 | *HSD17B12* | T/C | 0.72 | 0.54 | 0.96 | 0.027 | 1.000 |
| rs3768716 | *BARD1* | G/A | 1.41 | 1.03 | 1.92 | 0.031 | 1.000 |
| rs9295536 | *LINC00340* | C/A | 0.75 | 0.56 | 0.99 | 0.043 | 1.000 |
| rs2070096 | *BARD1* | C/G | 1.34 | 1.00 | 1.78 | 0.049 | 1.000 |

^a^ Minor allele/major allele; ^b^ Significant results after multiple correction are in bold.
