# Supplementary material for: Candidate Gene Association Analysis of Neuroblastoma in Chinese Children Strengthens the Role of LMO1
Source: PLoS One. 2015 Jun 1;10(6):e0127856. doi: 10.1371/journal.pone.0127856 (PMC4452511; doi:10.1371/journal.pone.0127856)
Supplement: S4 Table — (DOCX) [file pone.0127856.s005.docx]

**S4 Table.** Significant case-control haplotype frequency differences examined by two-sided χ^2^ test.

| Chromosome | Gene | Haplotypes | No. in case (%) | No. in control (%) | OR (95% CI) | *P* value | Adjusted *P* |
| --- | --- | --- | --- | --- | --- | --- | --- |
| CHR11 | *LMO1* | GCGCT | 72(14.7%) | 191(31.3%) | 0.38 (0.28-0.51) | 1.7×10^-10^ | 4.4×10^-9^ |
| CHR11 | *LMO1* | ATGGG | 156(32.0%) | 245(41.1%) | 0.67 (0.52-0.87) | 0.002 | 0.049 |
| CHR11 | *LMO1* | AGTA | 83(16.9%) | 65(10.6%) | 1.71 (1.21-2.43) | 0.002 | 0.071 |
| CHR11 | *LMO1* | GGAG | 65(13.4%) | 50(8.2%) | 1.73 (1.17-2.55) | 0.005 | 0.168 |
| CHR11 | *LMO1* | GATA | 204(41.8%) | 306(50.1%) | 0.71 (0.56-0.91) | 0.006 | 0.190 |
| CHR11 | *LMO1* | GTGG | 212(43.6%) | 316(51.8%) | 0.72 (0.57-0.92) | 0.007 | 0.216 |
| CHR11 | *LMO1* | AG | 253(51.8%) | 358(59.9%) | 0.72 (0.57-0.92) | 0.008 | 0.516 |
| CHR2 | *BARD1* | TGTCGAC | 86(17.6%) | 72(12.0%) | 1.58 (1.12-2.21) | 0.008 | 0.153 |
| CHR11 | *LMO1* | GTTAA | 188(38.5%) | 184(30.8%) | 1.40 (1.09-1.81) | 0.008 | 0.214 |
| CHR11 | *HSD17B12* | TTTGGCC | 2(0.4%) | 14(2.3%) | 0.18 (0.04-0-77) | 0.010 | 0.179 |
| CHR11 | *LMO1* | GG | 129(26.4%) | 119(19.9%) | 1.45 (1.09-1.92) | 0.011 | 0.690 |
| CHR11 | *HSD17B12* | CTCCGTA | 41(8.4%) | 80(13.1%) | 0.61 (0.41-0.90) | 0.013 | 0.232 |
| CHR11 | *LMO1* | CCATC | 108(22.0%) | 99(16.2%) | 1.47 (1.08-1.99) | 0.014 | 0.361 |
| CHR11 | *HSD17B12* | TCTGGCC | 385(78.9%) | 446(73.3%) | 1.36 (1.02-1.80) | 0.033 | 0.616 |
| CHR11 | *LMO1* | GCGCC | 57(11.7%) | 49(8.1%) | 1.51 (1.01-2.26) | 0.043 | 1.000 |
| CHR6 | *HACE1* | CA | 386(79.1%) | 451(73.9%) | 1.33 (1.01-1.77) | 0.045 | 1.000 |
